# Genome-wide analysis of rice dehydrin gene family: Its evolutionary conservedness and expression pattern in response to PEG induced dehydration stress

**DOI:** 10.1371/journal.pone.0176399

**Published:** 2017-05-01

**Authors:** Giti Verma, Yogeshwar Vikram Dhar, Dipali Srivastava, Maria Kidwai, Puneet Singh Chauhan, Sumit Kumar Bag, Mehar Hasan Asif, Debasis Chakrabarty

**Affiliations:** Council of Scientific and Industrial Research - National Botanical Research Institute (CSIR-NBRI), Lucknow, India; National Bureau of Plant Genetic Resources, INDIA

## Abstract

Abiotic stresses adversely affect cellular homeostasis, impairing overall growth and development of plants. These initial stress signals activate downstream signalling processes, which, subsequently, activate stress-responsive mechanisms to re-establish homeostasis. Dehydrins (DHNs) play an important role in combating dehydration stress. Rice (*Oryza sativa* L.), which is a paddy crop, is susceptible to drought stress. As drought survival in rice might be viewed as a trait with strong evolutionary selection pressure, we observed DHNs in the light of domestication during the course of evolution. Overall, 65 DHNs were identified by a genome-wide survey of 11 rice species, and 3 DHNs were found to be highly conserved. The correlation of a conserved pattern of DHNs with domestication and diversification of wild to cultivated rice was validated by synonymous substitution rates, indicating that *Oryza rufipogon* and *Oryza sativa* ssp. *japonica* follow an adaptive evolutionary pattern; whereas *Oryza nivara* and *Oryza sativa* ssp. *indica* demonstrate a conserved evolutionary pattern. A comprehensive analysis of tissue-specific expression of *DHN* genes in *japonica* and their expression profiles in normal and PEG (poly ethylene glycol)-induced dehydration stress exhibited a spatiotemporal expression pattern. Their interaction network reflects the cross-talk between gene expression and the physiological processes mediating adaptation to dehydration stress. The results obtained strongly indicated the importance of DHNs, as they are conserved during the course of domestication.

## Introduction

Plants effectively survive diverse and variable environments due to elasticity in their cellular metabolism, physiology, and development. However, extreme environmental conditions, such as the increased incidence of droughts, floods, and rising temperatures trigger extensive crop losses in most part of the world [1]. Abiotic stresses adversely affect cellular homeostasis, which, ultimately, impairs overall growth and fitness of plants. A plant’s behavioural responses to drought are complex and involve varied adaptive mechanisms. One such mechanism works *via* utilizing cellular mechanisms that produce an array of proteins that help with cellular adaptations to drought stress. Proteins encoded by the Late Embryogenesis Abundant (LEA) genes are expressed in response to water deficit [2]. LEA proteins have been identified in many plant species and are classified into six [2] or seven [3] classes. DHNs are a class of hydrophilic, thermostable stress proteins with a high percentage of charged amino acids, and they belong to Group II of the LEA family [4]. They supposedly carry out their function through membrane stabilization by acting as chaperones to prevent the aggregation and/or inactivation of proteins under dehydration [5]. DHNs are also called as RAB proteins because they are usually responsive to abscisic acid (RAB). It is already reported that when plants are exposed to various stresses related to cellular dehydration (e.g., drought, osmotic stress, salinity, and temperature), DHNs accumulate to high amounts in all vegetative tissues [6, 7, 8]. Several transgenic studies revealed that dehydrin gene expression has a positive effect on plant stress tolerance, including cold, drought and salinity. Kumar et al., [9] reported that *OsDHN1* gene overexpressed in rice (*Oryza sativa* L.) confers high tolerance to drought and salt stress. Similarly, Liu et al., [10] reported a positive correlation between *ShDHN* gene expression and cold and drought tolerance in tomato (*Solanum habrochaites* L.). Citrus (*Citrus unshiu* Marcov.) dehydrin gene, *CuCOR19* and maize (*Zea mays* L.) *ZmDHN2b* when overexpressed in tobacco showed tolerance to cold stress [11, 12]. In an another study, overexpressing dehydrin genes such as *ERD10*, *LTI30*, *RcDhn5*, and *DHN-5* in *Arabidopsis* plants showed tolerance to various abiotic stresses including freezing and salt [13, 14, 7]. Xu et al., [15] reported that overexpression of *Brassica juncea BjDHN2* and *BjDHN3* in tobacco resulted in higher tolerance to heavy metal stress. Therefore, it is well known that these proteins play an important protective role during cellular dehydration but their precise function remains unclear.

Rice (*Oryza sativa* L.) is susceptible to drought-induced stress as compared to other cereals [16] resulting in poor seedling vigor [17], fertility and adversely affecting the crop yield. The history of rice domestication has long been a subject of debate. The cultivated rice differs from wild-type progenitors in several ways and has lost many traits found in wild species, for example, loss of grain shattering and transition from perennial to annual crops [18]. Conscious selection by early farmers and unconscious selection due to agricultural practices or environments during long term domestication resulted in diversification of the cultivated rice gene pool across the geographic and climatic boundaries, leading to emergence of independent species, where its wild relatives could not grow at all [19]. Drought survival in rice might be considered a trait with strong evolutionary selection pressure. Therefore it would be interesting to examine the evolution of the drought-responsive protein (DHN) in rice in the light of domestication. Given the significance of DHNs in dehydration stress responses, the present study was performed in 9 species and 2 sub-species of *Oryza* to identify the presence and importance of DHNs during the course of domestication of rice. This study includes the six wild relatives of rice (*Oryza rufipogon*, *O*. *nivara*, *O*. *longstaminata*, *O*. *barthii*, *O*. *glumaepatula*, and *O*. *meridionalis*) and the cultivated *O*. *glaberrima* (African rice); the two subspecies of *Oryza sativa* (Asian), that is, *indica* and *japonica* with the AA genome [20]; and a wild variety from BB (*O*. *punctata*) [20] and FF (*O*. *brachyantha*) [21] genome to elucidate an almost precise picture of diversification, domestication, and evolution with respect to DHN. Of these, *O*. *rufipogon* is the species that is the closest to *O*. *sativa* ssp. *japonica* (hereafter mentioned as *japonica*) and is generally believed to be its progenitor, and *O*. *nivara* is believed to be the progenitor of *O*. *sativa* ssp. *indica* (hereafter mentioned as *indica*) [22].

## Materials and methods

### Identification of dehydrins and domain detection

To identify *DHN*s from 9 species and 2 sub-species of rice, a three- step approach was applied. Firstly, *Oryza sativa* ssp. *japonica DHN*s from database of Rice Genome Annotation Project (Release 7 of the MSU Rice Genome Annotation Project) were used as query sequences to search and retrieve *DHN*s from using BLASTP program[23]; applying e-value of 5 and considering the top hits with highest identity value for further analysis. Secondly, all possible sequence architecture for DHNs was searched using HMM profile of DHNs [24]. Finally, DHN sequences were searched in Plant Ensembl Database (www.plants.ensembl.org/) in each rice specie and sub-specie and then by name/function search. The protein sequences of the identified DHN members were then used as queries in multiple databases to ensure that no additional related genes were missed from the database. All sequences that satisfied the requirements were analysed using SMART database (http://smart.embl-heidelberg.de/), and the Conserved Domain Database of the NCBI (https://www.ncbi.nlm.nih.gov/Structure/cdd/cdd.shtml) to eliminate those genes that did not contain the conserved domains and motifs of the DHN family members.

### Multiple sequence alignment and phylogenetic analysis

Multiple sequence alignments were done on the amino acid sequences of identified DHNs using ClustelX version 2.0 [25] with default settings. Subsequently, MEGA7.0 software (http://www.megasoftware.net) [26] was used to construct an unrooted phylogenetic tree using the Maximum likelihood method with the bootstrap value of 1000.

### Chromosomal localization, whole genome duplication, gene structure and evolutionary analysis

Information about the physical locations of all identified *DHN* genes on chromosomes was mapped using CIRCOS tool (www.circos.ca/) [27]. Whole genome duplication (WGD) events are crucial for gene families and *DHN* genes were tested for their synteny conservation status, and the collinear blocks of identified *DHN*s were plotted using MCScanX tool [28]. Gene structure of *DHN* genes, showing their exon-intron boundaries and UTR regions, was generated using GSDS server (http://gsds.cbi.pku.edu.cn/) [29]. Interspecies evolutionary pattern was analyzed using PAL2NAL (www.bork.embl.de/pal2nal/) online tool which provided the rate of substitutions between the rice groups. An evolutionary time scale of rice species was generated using an online tool (www.timetree.org).

### Gene expression analysis

The *DHN* genes expression profile was retrieved from Rice Expression Profile Database (http://ricexpro.dna.affrc.go.jp), which provides expression from microarray analysis of tissues/organs encompassing the entire growth of the rice plant under natural field conditions and after treatment with various phytohormones [30].

### Gene motif and protein structural analysis

Promoter region sequences of all the retrieved *DHN* gene sequences were checked for the presence of important *cis*-regulatory elements using PLANTCARE (http://bioinformatics.psb.ugent.be/webtools/plantcare/html/). MEME *de-novo* motif detection tool (http://meme-suite.org) was used to find out novel sequence motifs in putative promoter region of *DHN*s. To check similarities and differences between the evolutionary connected rice groups, *in-silico* protein structural analysis was performed. Protein structures were modelled using PHYRE2 (www.sbg.bio.ic.ac.uk/~phyre) online server. Structures modelled by the server were then analysed for their structural properties using Chimera (https://www.cgl.ucsf.edu/chimera) 3D structure viewing tool.

### Plant growth, treatment and sampling

The rice cv. Nipponbare (*Oryza sativa* ssp. *japonica*) was germinated and allowed to grow for 5 day (D) at 26°C in dark and then transferred to Hewitt media for growth. After 10 D of growth, seedlings of uniform size and growth were treated with 10% PEG and 20% PEG under standard physiological conditions of 16 h light (115 μM quanta m^-2^ s^-1^) and 8 h dark photoperiod at 25 ± 2°C. Shoot and root samples were collected after 1, 3 and 7 D of treatment, and untreated samples were also collected on each day as control. All the samples were ground in liquid N_2_ and stored at -80°C for expression analysis.

For development specific expression patterns in various rice tissues/organs, i.e. seedling root (R), mature leaf (ML) and young/immature leaf (IL) and stages of reproductive development; young panicle (YP), anther (A) and gynaecium (G) were used for RNA extraction. Stages of seed development have been categorized according to days after pollination (DAP), middle and late globular embryo/ middle flowering stage (MF) after 5–10 DAP, and dormancy and desiccation tolerance embryo/after flowering stage (AF) after 21–25 DAP.

### RNA extraction, quantitative real-time PCR and network analyses

Total RNA was extracted from above samples by using RNeasy plant Mini Kit (QIAGEN, MD) and treated with *RNase* free *DNaseI* (QIAGEN, MD) according to manufacturer’s instructions. RNA integrity was checked by electrophoresis and quantified by using a NanoDrop^®^ ND-1000 UV-Vis spectrophotometer (Thermo Fisher Scientific). 2 μg RNA was reverse transcribed using oligo (dT) primers and Superscript II RT (Invitrogen, Rockville, MD, USA) into first- strand c-DNA in a 20-μL reaction as per manual instructions. Quantitative real-time PCR [31] was performed by Power SYBR Green PCR Master Mix (ABI, USA) using the ABI 7500 Fast Real-Time PCR Detection System (Applied Biosystems). The list of eight selected genes and oligonucleotide primers (Eurofins, India) for each gene used for development and drought related expression patterns are listed in S1 Table. Oligonucleotide primers for rice actin gene were used as the internal control for establishing equal amounts of cDNA in all reactions. The reactions were performed using the following cycle conditions: an initial 94°C for 2 min, followed by 30 cycles of 94°C for 30 s, 60°C for 30 s, and 72°C for 30 s, and the final 5 min extension at 72°C. The comparative ΔΔCt method was used to calculate the relative expression levels of different genes by using actin as the reference gene. All the experiments were repeated using three biological replicates. The data were analyzed by two way analysis of variance to confirm the variability and validity of results, and Duncan's multiple range test (DMRT) was performed for multiple comparisons of means. Significance levels were compared at p < 0.05 and the analyses were performed by SPSS 20.0 software. All *DHN*s from *japonica* were checked for their interaction network using STRING tool (http://string-db.org/).

## Results

### Identification, domain detection, multiple sequence alignment and phylogenetic analyses of dehydrins in *Oryza* species

Dehydrin proteins (DHNs) in *Oryza sativa* ssp. *japonica* were reported earlier as class 2 LEA proteins[4]. In the present study a total of 65 dehydrin genes (*DHN*) were identified from 11 *Oryza* groups (Table 1). The nomenclature of each DHN is made by following “Gene nomenclature system for rice” by McCouch and Cooperative [32]. We adopted the acronyms of *DHN1* to *DHN8*, in increasing order of their presence on their chromosome number.

**Table 1:** Structural features of identified *DHNs*.

Rice Cultivars	Protein Length (aa)	Name	Mwt (kDa)	pI
***Oryza barthii* (4)**				
OBART02G27280.1	290	ObaDHN1	30.9	5.9236
OBART11G12460.1	326	ObaDHN2	31.3	8.202
OBART11G12590.1	151	ObaDHN3	15.6	10.1527
OBART11G12600.1	447	ObaDHN4	45.2	9.7825
***Oryza brachyantha* (5)**				
OB02G34640.1	366	ObrDHN1	40.2	5.3197
OB11G19890.1	330	ObrDHN2	31.3	9.2766
OB11G19900.1	132	ObrDHN3	13.5	10.1673
OB11G19910.1	157	ObrDHN4	15.9	9.7211
OB11G19920.1	169	ObrDHN5	17.0	10.0721
***Oryza glaberrima* (6)**				
ORGLA01G0229900.1	654	OglaDHN1	71.2	7.3869
ORGLA02G0235300.1	290	OglaDHN2	30.9	5.9236
ORGLA11G0106700.1	326	OglaDHN3	31.3	8.202
ORGLA11G0107700.1	151	OglaDHN4	15.6	10.1527
ORGLA11G0107800.1	164	OglaDHN5	16.8	9.9226
ORGLA11G0107900.1	164	OglaDHN6	16.5	9.7765
***Oryza glumaepatula* (6)**				
OGLUM01G31840.1	612	OgluDHN1	66.3	6.7087
OGLUM11G12270.1	330	OgluDHN3	31.5	9.305
OGLUM11G12330.1	153	OgluDHN4	15.9	9.5319
OGLUM11G12340.1	164	OgluDHN5	16.7	9.7618
OGLUM11G12350.1	164	OgluDHN6	16.6	9.7765
OGLUM11G12360.1	185	OgluDHN7	18.7	10.0477
***Oryza meridionalis* (1)**				
OMERI02G26520.1	290	OmDHN1	30.9	5.9236
***Oryza nivara* (7)**				
ONIVA01G31820.1	654	OnDHN1	71.2	7.5921
ONIVA02G29800.1	292	OnDHN2	31.1	5.9236
ONIVA11G12140.1	326	OnDHN3	31.2	9.0859
ONIVA05G12830.1	151	OnDHN4	15.5	9.8049
ONIVA05G12790.1	176	OnDHN5	17.9	10.0476
ONIVA05G12840.1	84	OnDHN6	16.7	9.7618
ONIVA05G12780.1	164	OnDHN7	16.6	9.9399
***Oryza punctata* (7)**				
OPUNC01G27760.1	629	OpDHN1	68.3	7.7635
OPUNC02G24940.1	290	OpDHN2	30.8	6.0337
OPUNC11G10660.1	327	OpDHN3	31.4	9.0614
OPUNC11G10740.1	142	OpDHN4	14.9	10.0156
OPUNC11G10760.1	235	OpDHN5	23.8	10.2266
OPUNC11G10750.1	160	OpDHN6	16.5	9.776
OPUNC11G10780.1	168	OpDHN7	16.9	9.8538
***Oryza rufipogon* (7)**				
ORUFI01G30930.1	648	OrDHN1	68.3	7.7635
ORUFI02G28750.1	290	OrDHN2	30.9	5.9236
ORUFI11G13160.1	349	OrDHN3	33.8	9.7286
ORUFI11G13260.1	151	OrDHN4	15.6	9.805
ORUFI11G13290.1	160	OrDHN5	16.2	9.8538
ORUFI11G13280.1	276	OrDHN6	28.5	10.3513
ORUFI11G13270.1	59	OrDHN7	6.2	10.2178
***Oryza longistaminata* (7)**				
KN538697.1_FGP002	1279	OlDHN1	141.8	6.26
KN541601.1_FGP001	225	OlDHN2	24.4	5.5
KN539530.1_FGP003	142	OlDHN3	13.3	9.01
KN539940.1_FGP003	151	OlDHN4	15.6	9.4
KN539940.1_FGP004	119	OlDHN5	12.4	10.35
KN539940.1_FGP001	49	OlDHN6	4.9	9.77
KN542973.1_FGP002	109	OlDHN7	11.3	9.5
***Oryza sativa* ssp. *japonica* (8)**				
LOC_Os01g50700.1	653	OsjDHN1	71.0	7.07
LOC_Os02g44870.1	291	OsjDHN2	30.9	5.68
LOC_Os03g45280.1	93	OsjDHN3	10.4	6.7
LOC_Os11g26570.1	327	OsjDHN4	31.3	8.95
LOC_Os11g26750.1	152	OsjDHN5	15.5	9.13
LOC_Os11g26760.1	165	OsjDHN6	16.7	9.25
LOC_Os11g26780.1	165	OsjDHN7	16.5	9.27
LOC_Os11g26790.1	173	OsjDHN8	17.3	9.19
***Oryza sativa* ssp. *indica* (7)**				
BGIOSGA004279-PA	639	OsiDHN1	69.4	7.2237
BGIOSGA005869-PA	292	OsiDHN2	31.1	5.9236
BGIOSGA018448-PA	326	OsiDHN3	31.2	9.0859
BGIOSGA034054-PA	151	OsiDHN4	15.6	9.8049
BGIOSGA034053-PA	164	OsiDHN5	16.7	9.7618
BGIOSGA034051-PA	164	OsiDHN6	16.5	9.7921
BGIOSGA034052-PA	489	OsiDHN7	54.8	6.93

The identified genes were further checked for the presence of DHN domain. There are 15 reported architectures for DHN domain in plants, including monocots and dicots (http://pfam.xfam.org/family/PF00257#tabview=tab1). In our analysis we found only 4 domain architecture among 11 *Oryza* groups, *viz*. single small DHN, single large DHN, DHN-DNAJ, and DHN-DNAJ and DNAJ-X domains (S1 Fig). Exceptionally, *OsiDHN7* protein was observed with presence of FAR-1 domain.

The DHNs were further aligned by Clustal X software to confirm their sequence similarity. Interestingly, multiple sequence alignment of DHNs revealed the absence of K-seg in few rice DHN such as OnDHN6, OrDHN7, OlDHN3 and OlDHN6. During dehydration, the K-segments adopt α-helical conformation through the formation of class A2 amphipathic α-helix thus enhancing their amphipathic character in protein-protein or protein-biomembrane interactions [33]. It has been suggested by Koag et al [34] that these interactions between partly dehydrated surfaces of DHN molecules and other proteins and/or biomembranes, present the basis of dehydrin protective functions. OlDHN6 and OlDHN7 showed absence of S-seg which indicates the lack of phosphorylation of the S-segment which is associated with its transport to the nucleus and/or cation binding [33] (S2 Fig). DHNs varied in their length from 59 aa to 654 aa, molecular weight in the range of 13 to 71 kDa, and pI in the range of 5.6 to 10.3 (Table 1). Three *DHN* genes showed remarkable conservation in 7, 8 and 9 species, respectively, and were localised on chr1, 2 and 11, respectively. The *DHN* localised on chr 1 (*DHN1*) encoded a protein of 600 aa and had a representative single DHN domain with DNAJ and DNAJ-X domains. The *DHN* localised on chr 2 encoded for a protein of 290-293aa, and contained a single small DHN domain. The *DHN* localised on chr11 encoded a protein of 300–350 aa and had a single large DHN domain.

**Fig 1:**
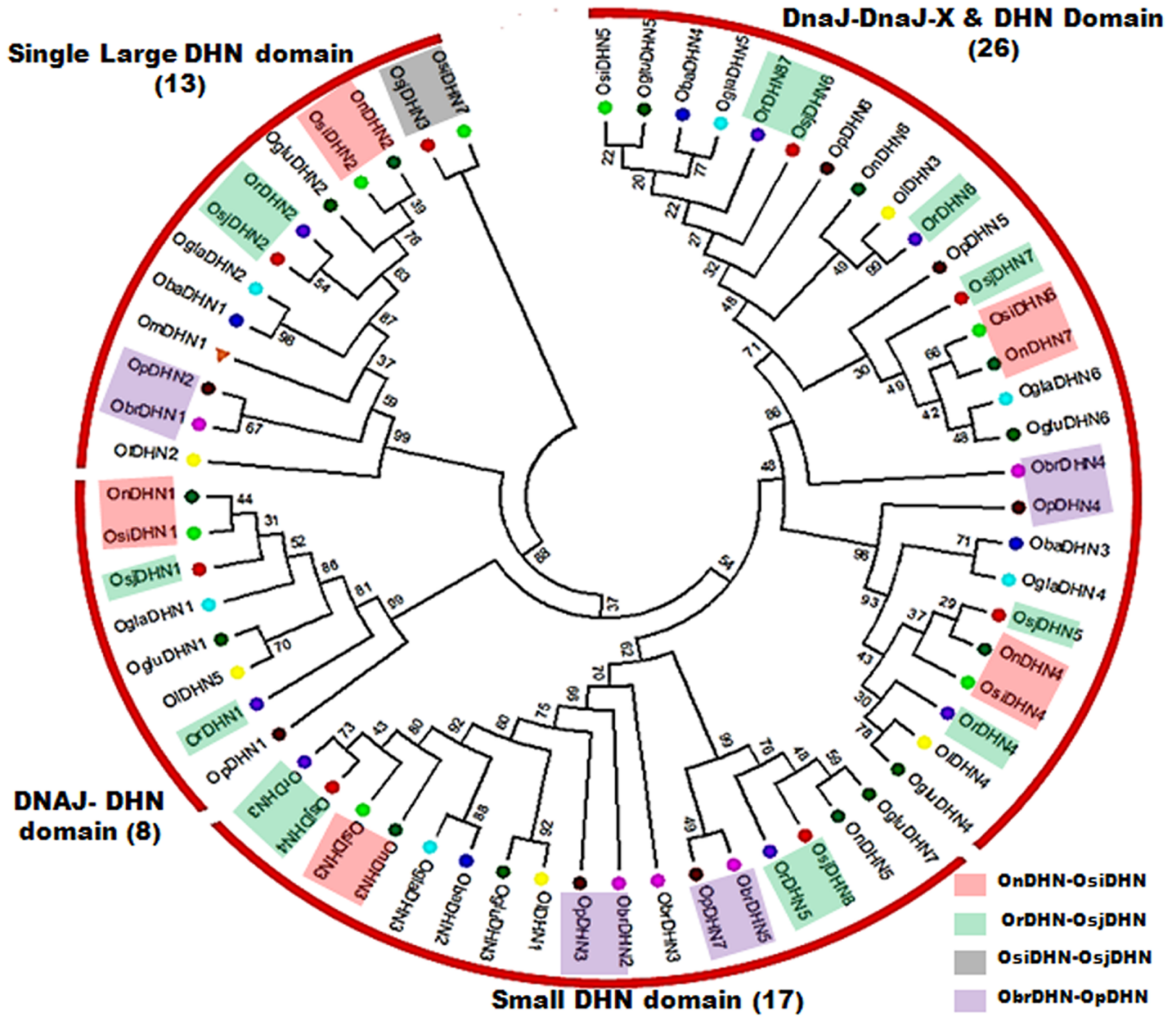
Phylogenetic analysis of the *DHN* gene in 11 rice species. The tree was calculated with MEGA 7.0 software.

To study the phylogenetic relationships among rice species we constructed an unrooted phylogenetic tree. Based on our phylogenetic results, the DHN’s could be divided into four groups, corresponding to four obtained DHN domain architecture in rice (Fig 1). Eight DHNs with DHNJ and DHN domain get clubbed into one clade, while 13 members containing single large DHN domain assembled in a single pool creating another clade. Similarly, 17 DHNs with single DHN domain forms another clade. 26 DHNs with DnaJ-DnaJ-X and DHN domain formed a clade bifurcating into two sub-clades. OnDHN2-OsiDHN2, OnDHN1-OsiDHN1, OnDHN7-OsiDHN6, OrDHN3-OsjDHN4 and OrDHN2-OsjDHN2 pairs were found to be proximally placed and had similar protein length, shared same clade with similar domain architecture pairs. Interestingly, the DHN’s of African domesticated variety *O*. *glaberrima* were also found proximal to both *japonica* and *indica*.

### Chromosomal localization, whole genome duplication, gene structure and evolutionary analysis

**Fig 2:**
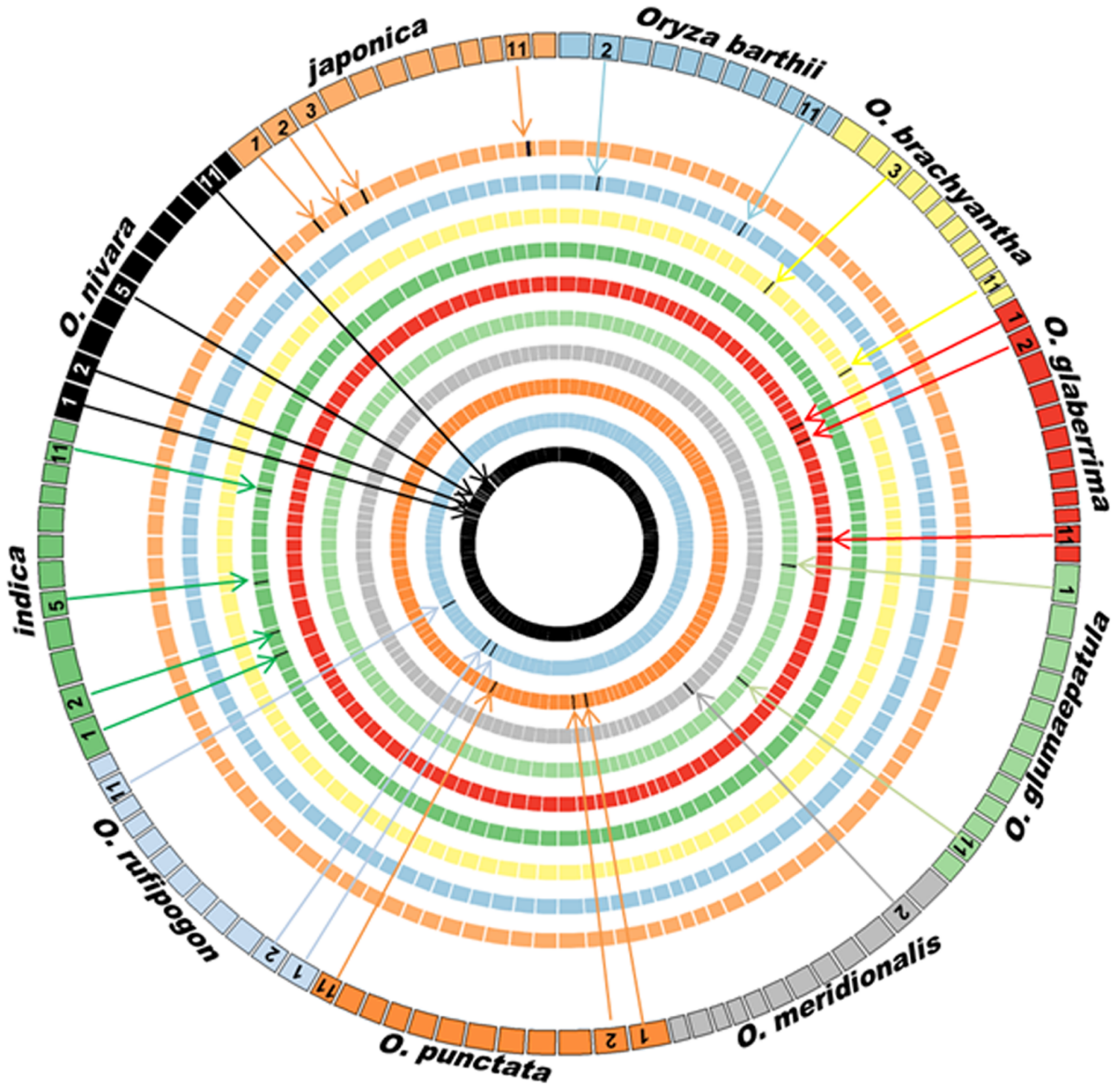
Chromosomal localisation of *DHN* genes in rice species; each colour and square representing specie and a chromosome respectively, and they are numbered clockwise.

**Fig 3:**
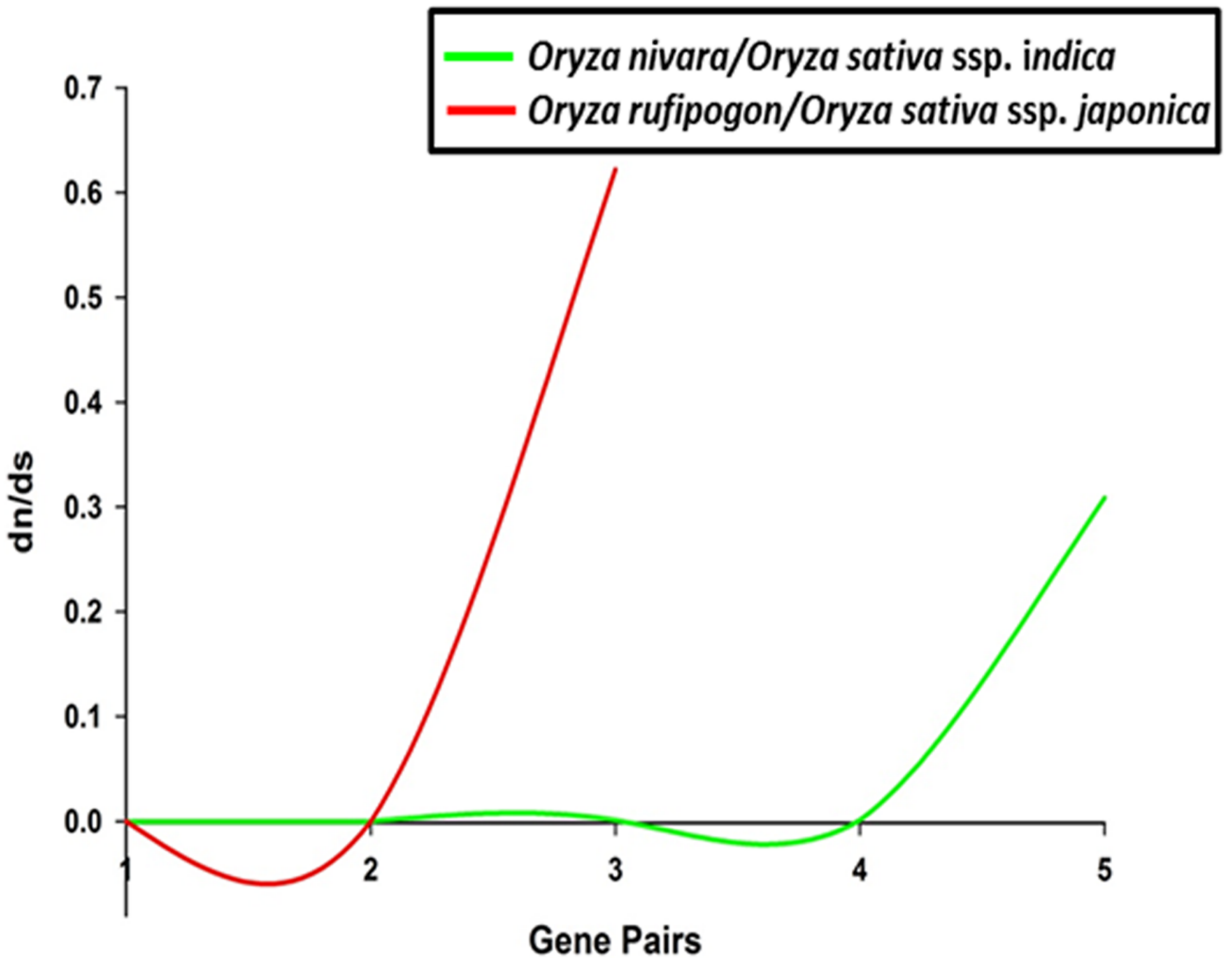
Divergence of *DHN* genes between orthologous gene pairs of N-I and R-J showing evolutionary substitution pressure on gene pairs.

**Fig 4:**
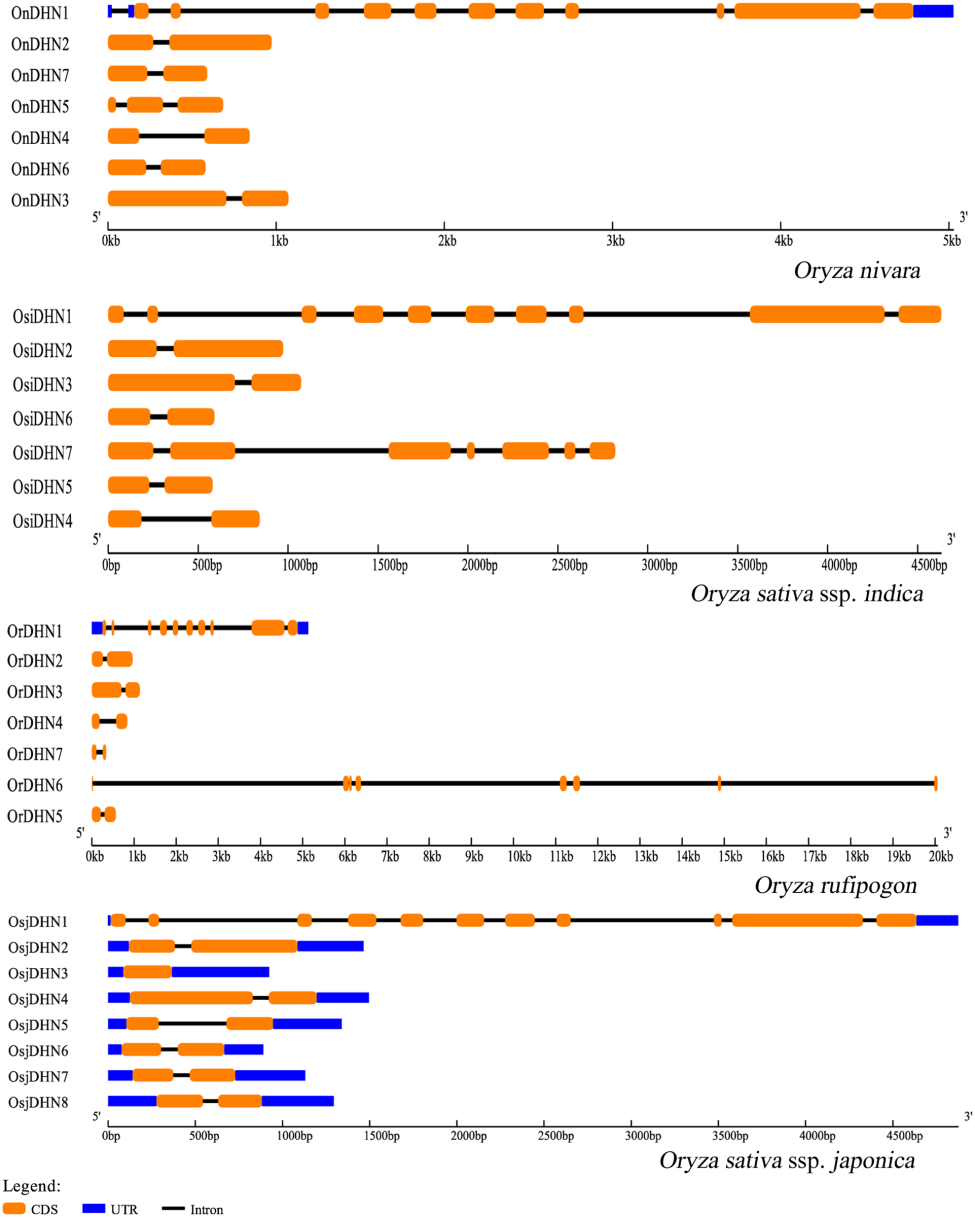
Gene structure of *O*. *nivara*-*indica* and *O*.*rufipogon*-*japonica* rice species showing their CDS, intron boundaries and UTR regions in orange black and blue colour respectively.

*DHN* genes were located on chromosomes 1, 2, 3, 5, and 11. The largest cluster was found on chromosome 11 while the others were majorly distributed on chromosome 1 and 2 (Fig 2). Gene duplication is recognized as an important requirement for the diversification of gene function. The identified *DHN* genes were checked for their synteny conservation status and the collinear blocks of *DHN*s were plotted. Overall, one duplication event was observed between chr 1–5 in *O*. *glumaepatula*, *O*. *barthii* and *O*. *nivara*. Two duplication events were observed in *O*. *glaberrima*, *O*. *brachyantha* and *O*. *punctata*, one between chr 1–5 and another between chr 8–9 (S3 Fig). The time tree was constructed for the approximate estimation of divergence in rice species. The time tree (S4 Fig) reflected that the Asian rice group diverted around 0.825 MYA (million years ago) whereas the African rice group diverted around 2.23 MYA. To check the evolutionary pressure, rate of substitution (synonymous and non-synonymous) was checked for all DHN sequences. We observed similar values for *ds* in Asian rice group, while their *dn* values varied (S2 Table). To further check evolutionary pressure on gene pairs we applied *dn*/*ds* ratio between orthologous gene pairs that has been calculated to analyse the synonymous and non-synonymous substitution rate for *O*. *nivara*-*indica* (hereafter mentioned as N-I) and *O*. *rufipogon*-*japonica* (hereafter mentioned as R-J) as they are evolutionary connected. Selection of ortholog gene pairs of rice groups was based on best hits of reciprocal blast. It could be inferred from 3 gene pairs that N-I followed evolutionary pattern to conserve the ancestral state, although 2 gene pair tends to follow adaptive pattern (Fig 3). The comparison of gene structure of *DHN* among N-I and R-J also support their independent evolutionary descent. In the case of R-J the CDS stretches are similar with the exception of absence of UTR region in *japonica*. Although N-I also shows similar gene structure for their orthologous *DHN* genes (Fig 4). The gene structures for the remaining rice species are given in S5 Fig.

### Gene expression

**Fig 5:**
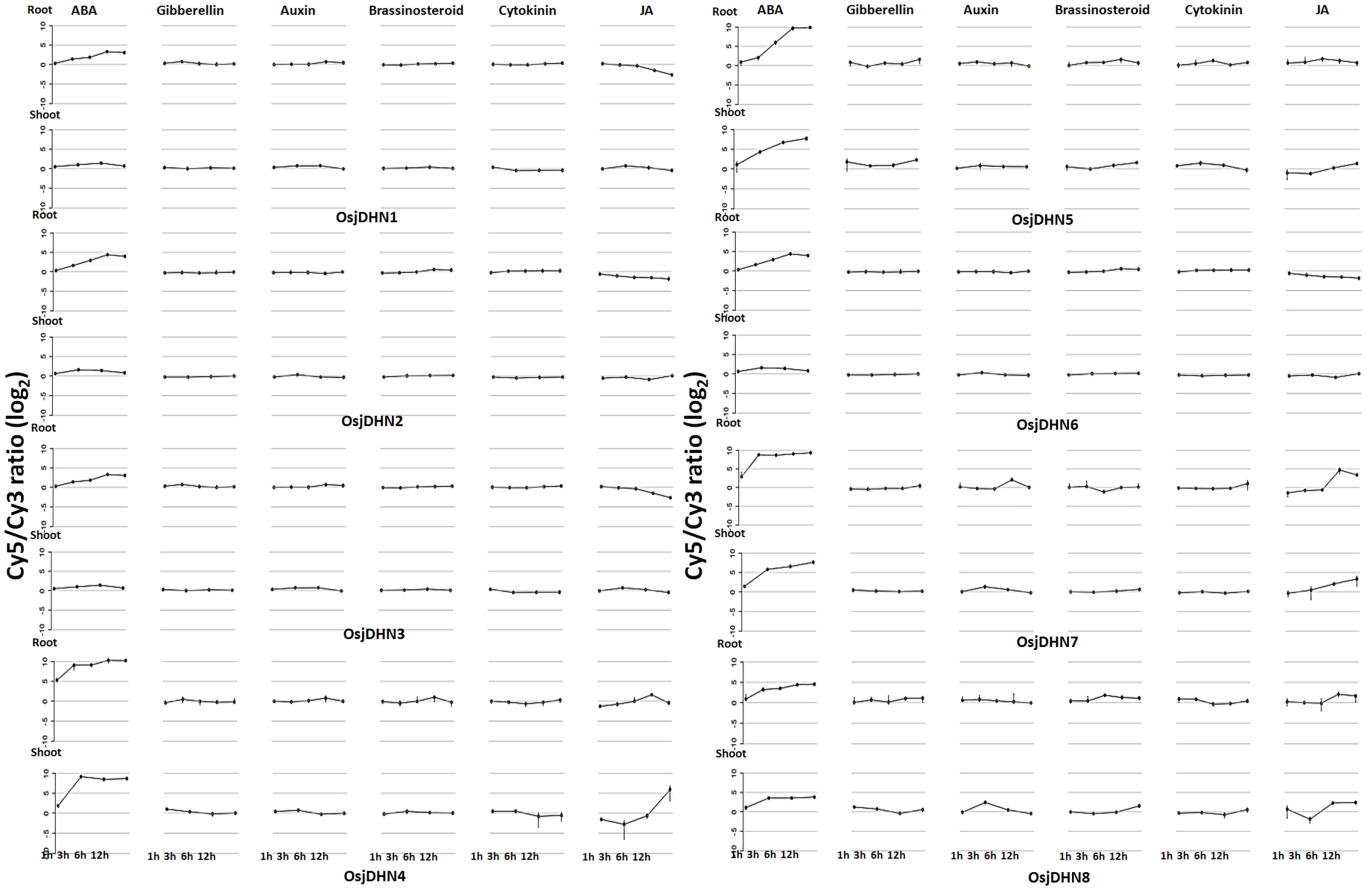
Expression profile of *DHN* genes in different hormonal conditions.

Expression profile of *DHN* genes has been retrieved and analysed under different hormone treatments which provided significant information regarding their involvement in hormonal signalling cascade. Expression data of *DHN*s under different hormonal conditions is taken from RiceXpro database (http://ricexpro.dna.affrc.go.jp/). The analysis revealed that, apart from phytohormone abscisic acid (ABA) and jasmonic acid (JA), other hormones do not alter expression of *DHN* gene. However, root expression data of *OsjDHN1* demonstrates the only instance of upregulation by cytokinin treatment. Dehydrin proteins play key roles in defences against stress, in either an ABA independent or -dependent manner. In our analysis, all genes were up-regulated by abscisic acid. In our study, the expression of *DHN* genes in response to JA demonstrated up-regulation in most of the genes for both root and shoot, though few genes did not show induction, while *OsjDHN1*, *OsjDHN2*, *OsjDHN3* in root showed down-regulation (Fig 5).

### Conserved motif search & structural analysis of DHN proteins

*cis*-regulatory elements are located upstream of genes and act as binding sites for transcription factors (TFs) and they have essential functions in determining the tissue-specificity or stress-responsive expression patterns of genes [35]. In order to understand potential functions of these *DHN* genes, 1000 bp regions upstream of the transcriptional start site were extracted and applied to identify *cis*-regulatory elements and other important motifs. Essential regulatory elements such as TATA box and CAAT box are present along with some other regulatory elements. A number of biotic stress-related *cis*-elements like EIRE, BOX W1, 5UTR Py-rich [33] were identified. Abiotic stress-related *cis*-elements were found in the putative promoters of *DHN* genes in rice, as for example: dehydration sensitive element (DRE), induction of CBF exspression region 2 (ICEr2), LTR motif (low temperature), Myb binding site (MBS) and sulfur-responsive element (SURE) [36]. Hormone-related *cis*-elements like- Abscisic acid responsive (CE-3, Motif IIb and ABRE)[37], auxin responsive (Aux RR), gibberellin-responsive (GARE, P Box) and Methyl jasmonate responsive (MeJA-RE, CGTCA) were found in the putative promoters of *DHN* genes in rice. The salicylic acid (SA) response is reflected by the presence of TCA elements [2]. These *cis*-elements are counted and classified in Table 2.

**Table 2:** Comparison of regulatory elements in 11 rice *DHN* promoter regions.

*cis*-element	Number of *cis*-elements											Function
	Oba	Obr	Ogla	Oglu	Ol	Om	On	Op	Or	Osj	Osi	
**ABRE**	1–3	3	0	3	1–3	0	3	3	0	3	3	Abscisic acid responsiveness
(GACACGTACGT)												
**DRE**	1	0	1	0	1	1	1–2	0	1	1	1	Drought and cold responsiveness
(ACCGAC)												
**LTR**	1	1–2	0	0	1	0	1–2	0	0	1–2	1–2	Low-temperature responsiveness
(CCGAAA)												
**TC-rich repeats**	0	0	1	1	0	1	0	1	1	0	0	Stress and defense responsiveness
(ATTCTCTAAC)												
**MeJA-RE**	2	1–3	1	1	2	1	1–3	1	1	1–3	1–3	MeJA-responsiveness
(CGTCA)												
**TCA element**	1	1–2	1	1	1	1	1–2	1	1	1–2	1–2	Salicylic acid responsiveness
(CAGAAAAGGA)												
**MBS**	0	0	1	1	0	1	0	1	1	1–3	1–3	MYB-binding sites
(CGGTCA)												
**Box-W1**	0	0	1	0	0	1	0	0	1	0	0	Fungal elicitor responsive
(TTGACC)												
**CGTCA**	2	3	1	1	2	1	3	1	1	3	3	Me-JA responsive
**TGACG**	3	1	1	1	3	1	1	1	1	1	1	Me-JA responsive
**GARE**	1	0	1	0	1	1	0	0	1	0	0	gibberellin-responsive
(TCTGTTG)												
**Aux-RR**	0	2	0	0	0	0	2	0	0	2	2	Auxin responsive
(GGTCCAT)												
**CE-3**	1	1	0	0	1	0	1	0	0	1	1	ABA & VP1 responsive
(GACGCGTG)												
**EIRE**	0	2	0	0	0	0	2	0	0	2	2	Elicitor responsive
(TTCGACC)												
**P-Box**	0	2	0	0	0	0	2	0	0	2	2	Gibberellin responsive
(CCTTTTG)												
**SURE**	0	0	1	1	1	0	1	0	0	1	1	Sulphur responsive element
(GAGAC)												
**PHR1-binding site**	1	0	1	0	0	0	1	0	1	1	1	Phosphorus starvation responsive
(GNATATNC)												
**5UTR Py-rich**	1	1	1	1	1	1	1	1	1	1	1	Biotic stress responsive
(TTTCTTCTCT)												
**Motif IIb**	0	0	0	0	0	0	1	0	0	0	0	ABA responsive
(CCGCCGCGCT)												
**GC motif**	1	0	1	0	0	0	1	0	1	1	1	Enhancer like, anoxic specific inducibility
(AGCGCGCCG)												
**ARE**	1	0	1	1	1	0	0	1	1	0	0	Essential for anaerobic induction
(TGGTTT)												
**MYCR**	1	1	0	1	1	1	1	1	1	1	1	Water deficit, ABA responsive
(CACATC)												

**Fig 6:**
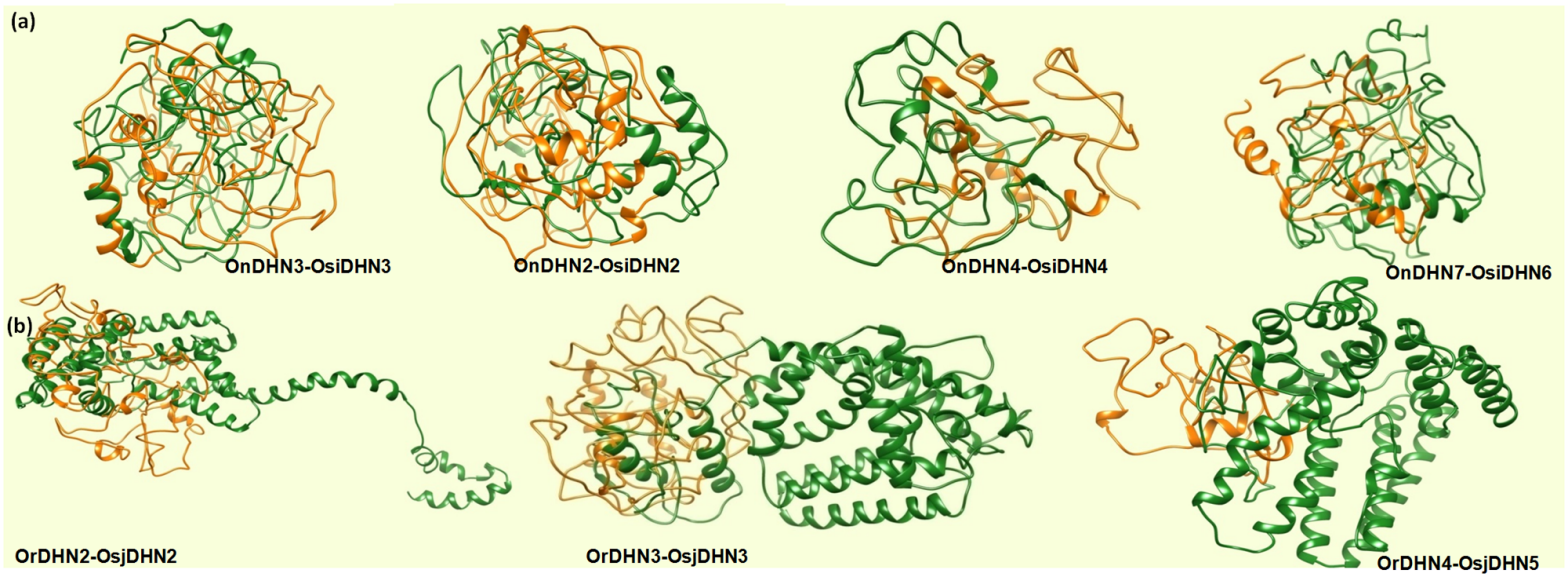
Structural prediction of *DHN* genes, *in-silico* structural analysis of orthologous gene pairs of N-I and R-J. Proteins in green from (a) *O*. *rufipogon* and (b) *O*. *nivara* and proteins in orange indicate the structure of DHN proteins from (a) *japonica* and (b) *indica*.

The structure of each DHN protein was analysed and modelled using PHYRE2 web server showed abundance of loops in the secondary structure of DHNs in *japonica*, indicative of divergence, whereas the secondary structure of *O*. *rufipogon* comprised mainly of α helices (Fig 6a). Interestingly, in the case of N-I, abundance of loops was observed in all orthologous pairs (Fig 6b).

### Expression profiles of *DHN* genes in various tissues, organs, and developmental stages

**Fig 7:**
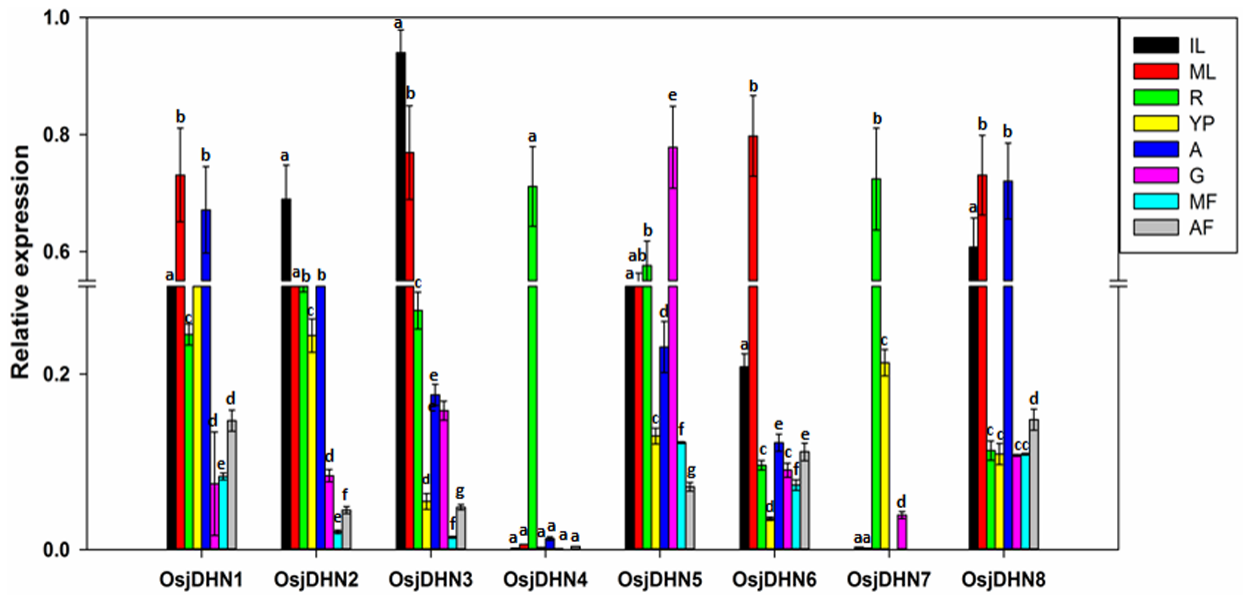
Relative expression analysis of tissue- or organ-specific *DHN* gene expression profiles in rice species. R, roots, S, mature leaf (ML), immature/young (IL) androecium (A) gynoecium (G), young panicle (YP) middle flowering stage (MF) and after flowering stage (AF.) The expression levels of genes are presented using fold-change values transformed to Log_2_ format.

qRT-PCR analysis of various tissues, organs and developmental stages indicated high variability in transcript abundance of *DHN* genes in rice (Fig 7). Majority of these genes, except *OsjDHN4* and *OsjDHN7*, showed relatively high expression in vegetative organs like root (R), mature leaf (ML), immature/young leaf (IL) and reproductive organ like androecium (A). However, the gene showed lower transcripts in gynoecium (G), young panicle (YP), middle stage of flowering and after stage of flowering. These findings indicated spatio-temporal expression characteristics of *DHN*s. Two genes viz. *OsjDHN4* and *OsjDHN7* showed high expression only in root, indicating their possible function in dehydration tolerance. All *DHNs* except *OsjDHN1* and *OsjDHN4* accumulated more in leaves (IL and ML). Roots also accumulated higher amount of DHNs, however *OsjDHN*6 and *OsjDHN8* expression was lesser in comparison to all the *DHNs* in root tissue. *OsjDHN4* specifically accumulates in roots in comparison to all other *DHNs* and all other tissues.

### Expression profiles of *DHN* genes under PEG treatment in *japonica* rice and gene interaction network

**Fig 8:**
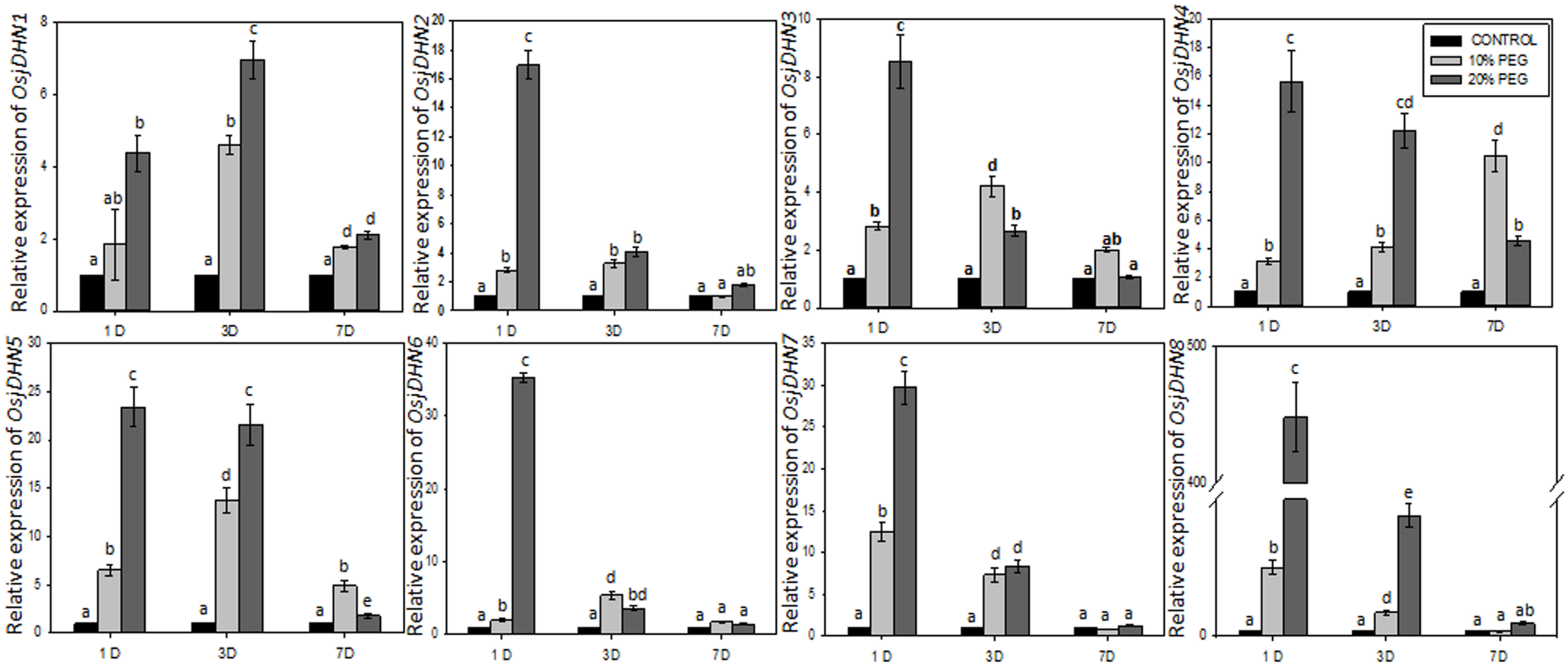
Relative expression analysis of *DHN* genes in shoots. The expression levels of genes are presented using fold-change values transformed to Log_2_ format compared with control. All the values are means of triplicate ± SD. Values marked with similar letters are not significantly (Duncan’s test: p < 0.05) different in a particular tissue/treatment.

The expression analysis of 8 genes in shoots, on day (D) 1 and moderate (10%PEG) stress condition resulted in nearly 3 fold increase in expression of *OsjDHN2*, *OsjDHN3*, and *OsjDHN4*. However, *OsjDHN7* and *OsjDHN8* demonstrated nearly 12 fold increase in transcript abundance under same condition. During severe (20%PEG) drought stress, all *DHN* genes were upregulated with *OsjDHN8* showing 448 fold increase in expression pattern. *OsjDHN5*, *OsjDHN6 and OsjDHN7* demonstrated almost 30 fold increase in their transcript abundance. Thus, in shoots, on D1 more DHNs expressed significantly during 20% PEG induced drought stress. On D3, *OsjDHN4*, *OsjDHN5* and *OsjDHN7* showed 10 fold greater expressions during 20% PEG treatment as compared to 10% PEG treatment. However, other *DHN* genes expressions were almost similar under moderate and severe dehydration stress. However, on D7, only *OsjDHN4* and *OsjDHN5* showed transcript abundance during moderate stress conditions; whereas only *OsjDHN4* was upregulated during 20% PEG induced dehydration stress (Fig 8).

**Fig 9:**
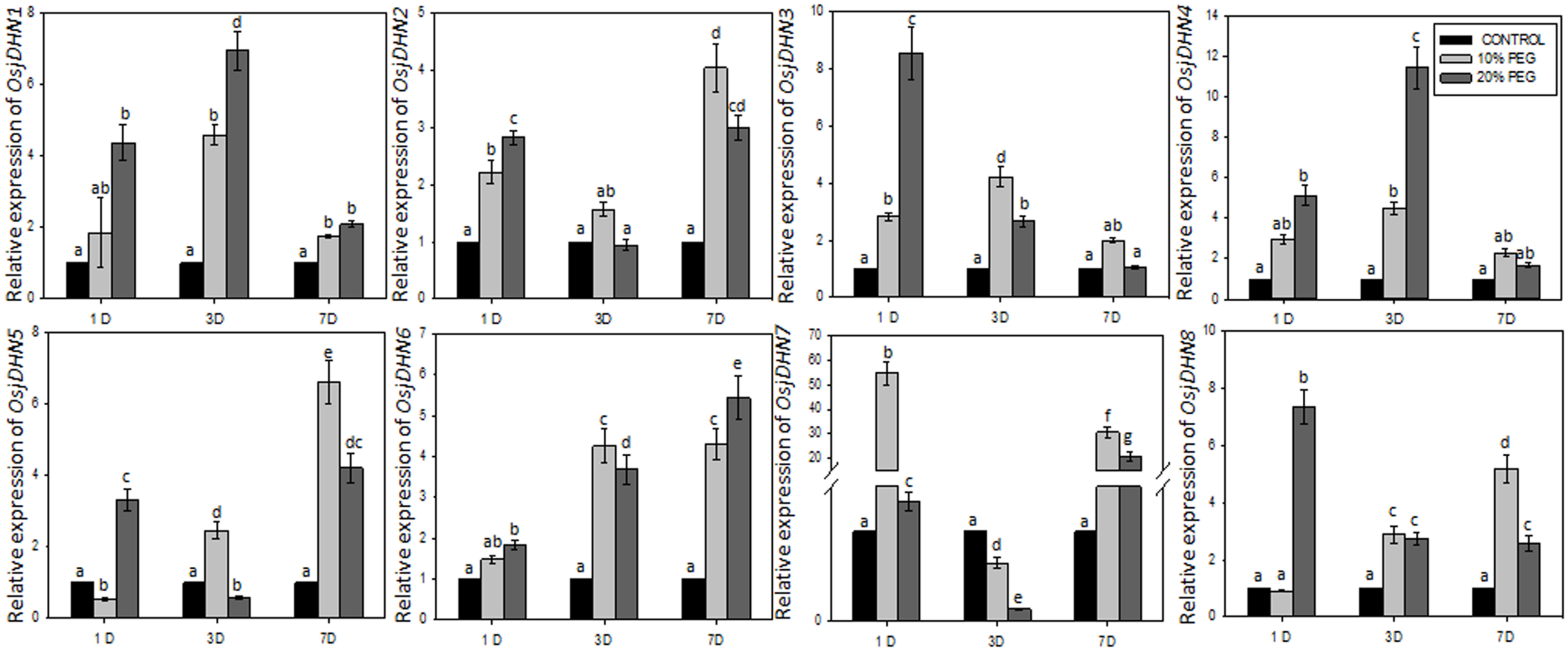
Relative expression analysis of *DHN* genes in roots for the response patterns to moderate (10% PEG) and severe drought (20% PEG) conditions. The expression levels of genes are presented using fold-change values transformed to Log_2_ format compared with control. All the values are means of triplicate ± SD. Values marked with similar letters are not significantly (Duncan’s test: p < 0.05) different in a particular tissue/treatment.

In roots, on D1, only *OsjDHN*7 exhibited around 55 fold increase in expression level during moderate stress conditions; whereas *OsjDHN8* expression levels were high during 20% PEG treatment. On D3, expression level of *OsjDHN4* was around 6 fold greater during 20% PEG treatment as compared to 10% PEG treatment. However, other *DHN* genes expressions were almost similar under moderate and severe dehydration stress. On D7, almost all DHNs were upregulated, expect for *OsjDHN1* and *OsjDHN4*; interestingly, these two genes were upregulated on D3 (Fig 9). Unexpectedly, the transcript abundance on D3 and 7 was reversed in roots and shoots, thus signifying the spatiotemporal expression pattern of *DHN*s during drought conditions. On D7, *OsjDHN2*, *OsjDHN5*, *OsjDHN6*, and *OsjDHN8* showed nearly 4~5 fold higher transcript abundance; *OsjDHN7* demonstrated 30 fold higher expression during 10% PEG treatment. During 20% PEG treatment *OsjDHN4 and OsjDHN7* showed 11 and 21 fold greater expression, while *OsjDHN5* and *OsjDHN6* showed nearly 4~5 fold higher transcript abundance (Fig 9).

*OsjDHN7* had ABRE, DRE, LTR rich regulatory elements in their promoter region. *cis*-element of *OsjDHN5* was exclusively rich in ABA- responsive elements. However, both ABRE and DRE were absent in *OsjDHN1*, other regulatory elements like MYB, TC rich repeats and 5’ UTR Py-rich were present (S3 Table).

**Fig 10:**
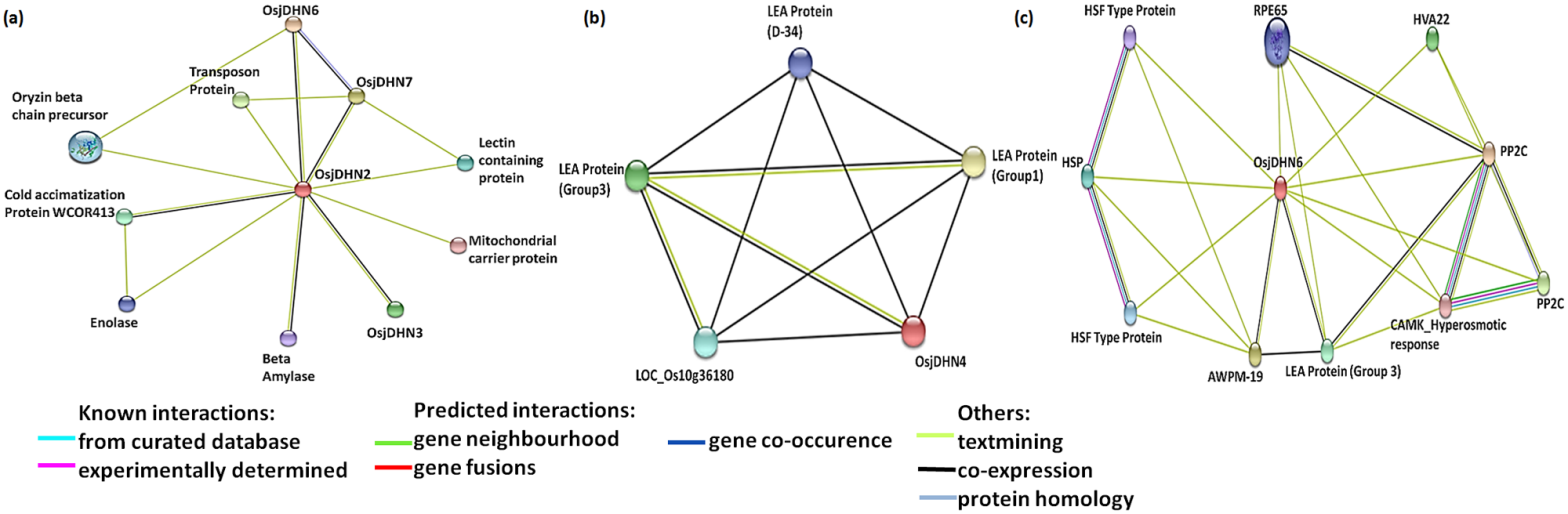
Gene interaction network of *OsjDHN7* (a), *OsjDHN4* (b), and *OsjDHN6* (c).

The gene interaction network of *OsjDHN2*, *OsjDHN4*, *OsjDHN6*, and *OsjDHN7* further demonstrates the interaction pattern where the network involves other *DHN*s. *OsjDHN2* networks also involve *OsjDHN3*, *OsjDHN6*, *OsjDHN7* and cold acclimatization protein (WCOR413) and other metabolic pathways (Fig 10a). On the other hand, *OsjDHN4* requires the presence of *LEA* genes in their interactional network (Fig 10b). In the case of *OsjDHN6*, we found the presence of *OsjDHN7*, which further interacts with LEA proteins (Fig 10c). *OsjDHN6*, showed an interaction with HSPs, an abscisic acid-induced protein (HVA22) and cold shock-resistant genes (AWPM-19).

## Discussion

Dehydrins are protective and ubiquitous proteins that are found in plants undergoing osmotic stress, for example, cold, high salinity, and drought. Domestication, being a unique form of biological evolution, may decrease the overall fitness of plants in the wild but increase adaptability when completely dependent on humans for their survival, leading to a loss of certain traits. Drought stress is posed by adverse environmental conditions, and surviving drought could be viewed as a trait that is more related to wild species and diversification. Complete and accurate identification of *DHN* genes in wild and domesticated species is an essential step towards further evolutionary and functional studies of the gene family. By definition, all DHNs contain, at least one copy of a highly conserved sequence, the K-seg, and may or may not include other conserved sequences, the S-seg and the Y-seg [38]. On the basis of the presence of the conserved sequences, we identified 65 *DHN*s in eight wild, one domesticated species and two rice sub-species. The absence of K-seg in DHN such as OnDHN6, OrDHN7, OlDHN3 and OlDHN6 was observed. One such DHN, lacking complete K-segment has been also been reported by Perdiguero et al [39]However, these four proteins show high overall similarity in domain architecture and homology with typical dehydrins, especially in N-terminal region, S-segment and Y-segment, which leads us to still consider them as DHNs.

The presence of DHNs in all these diverse rice species is indicative of their significance, as they have not been excluded during the course of domestication. The protein domain architecture is related to evolutionary dynamics [40], and the presence of only a4-domain architecture among *Oryza* species and sub-species indicates their functional specificity for rice groups. Three *DHN* genes with conserved chromosomal localization in 7, 8, and 9 species signifies the importance of DHNs in domestication as well as evolution of rice, and thereby emphasizing their significance in the dehydration cascade in different *Oryza* species.

The phylogenetic analysis indicated protein pattern similarity between *O*. *rufipogon* with *japonica* and *O*. *nivara* with *indica*. The phylogenetic tree supports the independent origin of *japonica* and *indica* from *O*. *rufipogon* and *O*. *nivara* respectively, as the *DHN* of these species are paired and share the same clade with their diverged wild progenitors. OrDHN1-OsjDHN1 is in one clade but is distantly placed in the phylogenetic tree, signifying the adaptive gain during the course of evolution. However, the proximity of African domesticated rice- *O*. *glaberrima*, with Asian domesticated rice—*japonica* and *indica*, indicates convergent yet independent selection of common genes during two geographically distinct domestication processes [41]. Few OnDHN-OsiDHN and OrDHN-OsjDHN pairs were found to be in one clade but not paired, as they must have probably lost one of their conserved stretches (K, Y, or S) but were able to maintain their functionality. This again supports the evolutionary adaptation of *DHNs* during the course of domestication. Evolutionary proximity was observed between the BB genome representative- *O*. *punctata* and the FF genome representative- *O*. *brachyantha*, where the *DHN* representatives of both species were present in one clade, indicating the evolutionary relationship between BB and FF genomes. Gene duplication is recognized as one of the important requirements for diversification of gene function [42]. Expansion of the DHN family has generally occurred through tandem and whole-genome duplication (WGD) events. For example, 3 genes each from tandem duplications (TD) and WGD were reported in *Arabidopsis* [43], resulting in an increase of 6 *DHN* genes. Similarly in poplar, WGD and TD events were responsible for an increase of 3 and 2 *DHN* genes [44], respectively. Three TD events have been reported in *japonica* rice [4]. Overall, two duplication events were observed, one between chr 1–5 and another between chr 8–9, except for *O*. *nivara*, no *DHN* was found to be present on either chr 5, 8 or 9. As mentioned previously, *DHN1* encodes a protein with single DHN domain with DNAJ and DNAJ-X domains. The duplicate genes found on chr 5, 8 and 9 were cross-checked for domain detection and we found that collinearity on chr 5, 8 and 9 was observed due to the presence of larger stretch of DNAJ and DNAJ-X domain and not on the basis of DHN domain. Thus it could be inferred that, despite of the presence of segmental duplication event, no *DHN* exhibit synteny conservation. The result of time of divergence of Asian rice group and their substitution analysis implies that the evolution of DHN proteins of *indica* is connected to *O*. *nivara* and demonstrates a conserved evolutionary pattern, which is also supported by the gene structure of the *DHN* genes in both species. On the other hand, the substitution pattern of R-J *DHN* pairs shows an adaptive evolutionary pattern. It has been already reported that *japonica* grows in a much varied environment as compared to *indica*, and the former might have gained divergent features as compared to *O*. *rufipogon* during the course of domestication. The gene structure analysis of *DHN*s in N-I and R-J in our study is also in agreement with the independent evolutionary descent of *japonica* and *indica*.

Dehydrins are major key players in providing defences against stress. However, whether they are utilized in a hormone-independent or -dependent manner is still unclear. For example, *Arabidopsis* At1g54410 is induced by cold but not by abscisic acid [43]. In our analysis, all *DHN* genes were upregulated by this hormone, indicating their possible involvement in ABA-dependent pathways. It has also been reported that many genes involved in jasmonic acid (JA) biosynthesis and signalling are induced by drought and cold treatment [45]. Similar results have been observed when the overexpression of *DHN5* in wheat resulted in down-regulation of genes encoding 3 members of JAZ (jasmonate- ZIM domain) proteins, which are negative regulators of JA signalling. Thus, differential expression of JA might depend on the complex inter-regulation of the signalling cascade. *cis*-regulatory elements, being located upstream of genes and acting as binding sites for TFs, are instrumental in determining the tissue specificity or stress-responsive expression patterns of genes [35]. When taken together, all of the putative regulatory elements identified within rice species and sub-species, the promoters had the most diverse collection of putative *cis*-regulatory elements, including several involved in stress response, like MBS involved in drought inducibility in *OsGATL*s in rice [46]; drought and salinity together or alone lead to enrichment of 2 *cis* elements ABRE and DRE [47]; and hormone signalling like CE-3 and Motif IIb [37]. Presence of biotic stress responsive elements like Box-W1 and EIRE also indicates the diverse activity of DHNs.

DHNs being intrinsically disordered proteins (IDPs) with ~ 0.7–5% of α-helices [48], these unstructured states assist the protective functions of DHN proteins, resulting in their functional versatility [49]. Proteins belonging to the same family may contain a similar active domain but due to their conformational changes, they may show different behaviours. Our results demonstrated the change in folding pattern of DHNs from *O*. *rufipogon* (comprised mainly of α-helices) have evolved into IDPs (as seen in *japonica*), providing functional benefits, such as an increased interaction surface area and conformational flexibility to interact with several targets. However, *indica* and *O*. *nivara* exhibited a similar structural prediction. This is in corroboration with the results obtained by synonymous and non-synonymous substitution rate analysis.

The expression analysis indicated a contrasting expression pattern between vegetative and reproductive organs. *OsjDHN3* showed high expression only in leaves (IL and ML). One possibility is that the high osmotic potential is generated in the cytoplasm of guard cells of leaves during stomatal opening which probably promote accumulation of DHNs in these tissues. The high osmotic potential in the cytoplasm of open guard cells promotes water stress on the nucleus. Thus, DHNs may have a role in protecting this compartment from dehydration stress. This is in agreement with the postulated protective function for these proteins during osmotic stress caused by either drought, salinity or freezing [50]. Two genes, that is, *OsjDHN4* and *OsjDHN7*, showed high expression only in roots; thus, possibly involved in the water level balance in the roots. As roots are the primary target for dehydration stress DHN accumulation in roots are in accordance with their protective role during dehydration stress. *OsjDHN*5 was the only gene exhibiting transcript abundance in gynoecium. However all genes except *OsDHN4* showed transcript abundance in reproductive tissue. These *DHN*s may have protective role against the desiccation or dehydration stress during the development of seeds. Roots are generally believed to be the main organs involved in drought stress, although our study showed a greater induction rate of *DHN* genes in shoots. Interestingly, a unique pattern was observed in gene induction by dehydration in both the tissues. In shoots, higher expression levels of *OsjDHN*s were observed during initial phase (D1) with moderate stress. However, *OsjDHN8* was found to be upregulated by 448 fold during severe stress conditions. Contrarily, during severe and prolonged dehydration stress only *OsjDHN4* was upregulated by 10 fold. In roots, almost all *OsjDHN*’s were upregulated during moderate stress. *OsjDHN7* demonstrated 30 and 20 fold higher expression during moderate and severe stress, respectively. *OsjDHN7* was the only gene with 12 fold higher transcript abundance on D3 during severe stress. The transcript abundance on D3 and 7 was reversed in roots and shoots, thus signifying the spatiotemporal expression pattern of *DHN*s during dehydration stress. The expression analysis exhibits the importance of *OsjDHN1*, *OsjDHN4* and *OsjDHN7* in imparting PEG induced dehydration tolerance. Interestingly, *OsjDHN1* and *OsjDHN4* are among 3 DHNs that were found to be conserved on the basis of their chromosomal localization, protein length and domain architecture. Also, the *OsjDHN1* expression pattern was similar in both tissues. Similar results were reported by Kumar et al. [9], stating that *OsDhn1*-overexpression in transgenic rice plants (*OsDhn1-OXs*) shows enhanced drought and salt stress tolerance. The dehydration tolerance could be attributed to presence of abiotic stress related *cis*-regulatory elements, especially ABRE and DRE motifs. Due to the presence of ABRE motif, it could be concluded that all *OsjDHN*s (except *OsjDHN1*) expression is probably regulated by ABA dependent signalling pathways. Various other hormone related *cis* elements like CGTCA and TCA elements were found and they are in corroboration with our gene expression analysis. The gene interactions of *OsjDHN2* involve *OsjDHN3*, *OsjDHN6*, and *OsjDHN7*; thus, it could be possible that these three genes require *OsjDHN2* as a signal for their response. The following network is also reflected by the expression profiles of PEG-treated, roots and shoots, where the expression pattern of *OsjDHN3*, *OsjDHN6*, and *OsjDHN7* is similar to that of *OsjDHN2*. On the other hand, *OsjDHN4* requires the presence of *LEA* genes in their interactional network. The expression profile of *OsjDHN6* also indicated their role in signalling the expression of *OsjDHN7*, which further interacts with LEA proteins and illustrates a specific pattern in the shoot expression profile in PEG-treated plants. *OsjDHN6*, which was highly expressed during PEG treatment, showed an interaction with HSPs and cold shock-resistant genes (AWPM-19), also indicating its specific role in combatting dehydration caused due to low temperature stress.

## Conclusion

In summary, a total 65 DHNs were identified in *Oryza*, 9 species and 2 sub-species. This study provides an insight into the evolutionary association of DHNs. The phylogeny, domain detection, and MSA exhibited useful information about conserved DHNs, indicative of their functional conservation in varied rice species. Structural analysis and evolutionary pattern supported the independent origin of *japonica* and *indica* rice varieties. We also focused on the response patterns of *DHN*s genes in drought conditions, and their interaction network revealed their mode of action and their signalling properties. Our result advocates DHNs as conserved proteins during the course of domestication/evolution of rice, and they play an important role in combating dehydration stress. Also, the results provide a base for further functional and evolutionary studies of the *DHN* gene family in rice and other plants.

## Supporting information

S1 Fig Observed DHN domain architecture in 11 rice species. (TIF)

S2 Fig Multiple sequence alignment of the DHN amino acid sequences. The alignment was performed using ClustalX. The conserved K Y and S segments are highlighted.
(TIF)

S3 Fig Gene structure of rice cultivars showing their CDS, intron boundaries and UTR regions in orange black and blue colour respectively. (TIF)

S4 Fig Synteny conservation of DNAJ and DNAJ-X domain in rice species. Each red line indicates one duplication event.
(TIF)

S5 Fig Tree representation of rice species on the basis of evolutionary timescale. (TIF)

S1 Table Forward and reverse primers used for qRT PCR analysis. (DOC)

S2 Table Synonymous and non-synonymous substitution analysis in rice species. (DOC)

S3 Table Comparison of regulatory elements in 8 *DHN*s of *Oryza sativa* ssp *japonica*. (DOC)
