# Supplementary figures and images for: Genome-wide analysis of rice dehydrin gene family: Its evolutionary conservedness and expression pattern in response to PEG induced dehydration stress
Source: PLoS One. 2017 May 1;12(5):e0176399. doi: 10.1371/journal.pone.0176399 (PMC5411031; doi:10.1371/journal.pone.0176399)

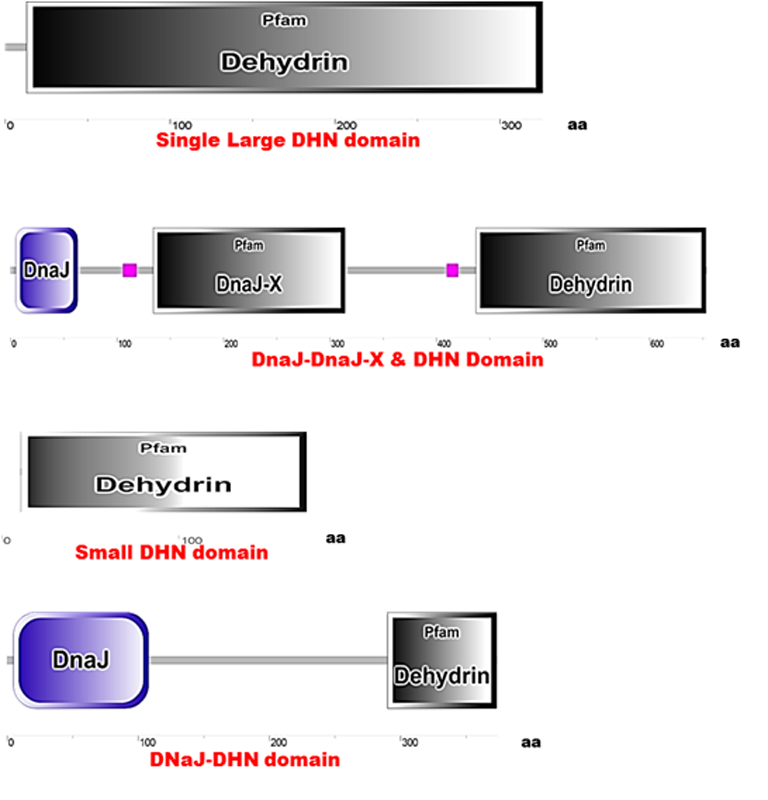

Supplement: S1 Fig — (TIF) [file pone.0176399.s001.tif]

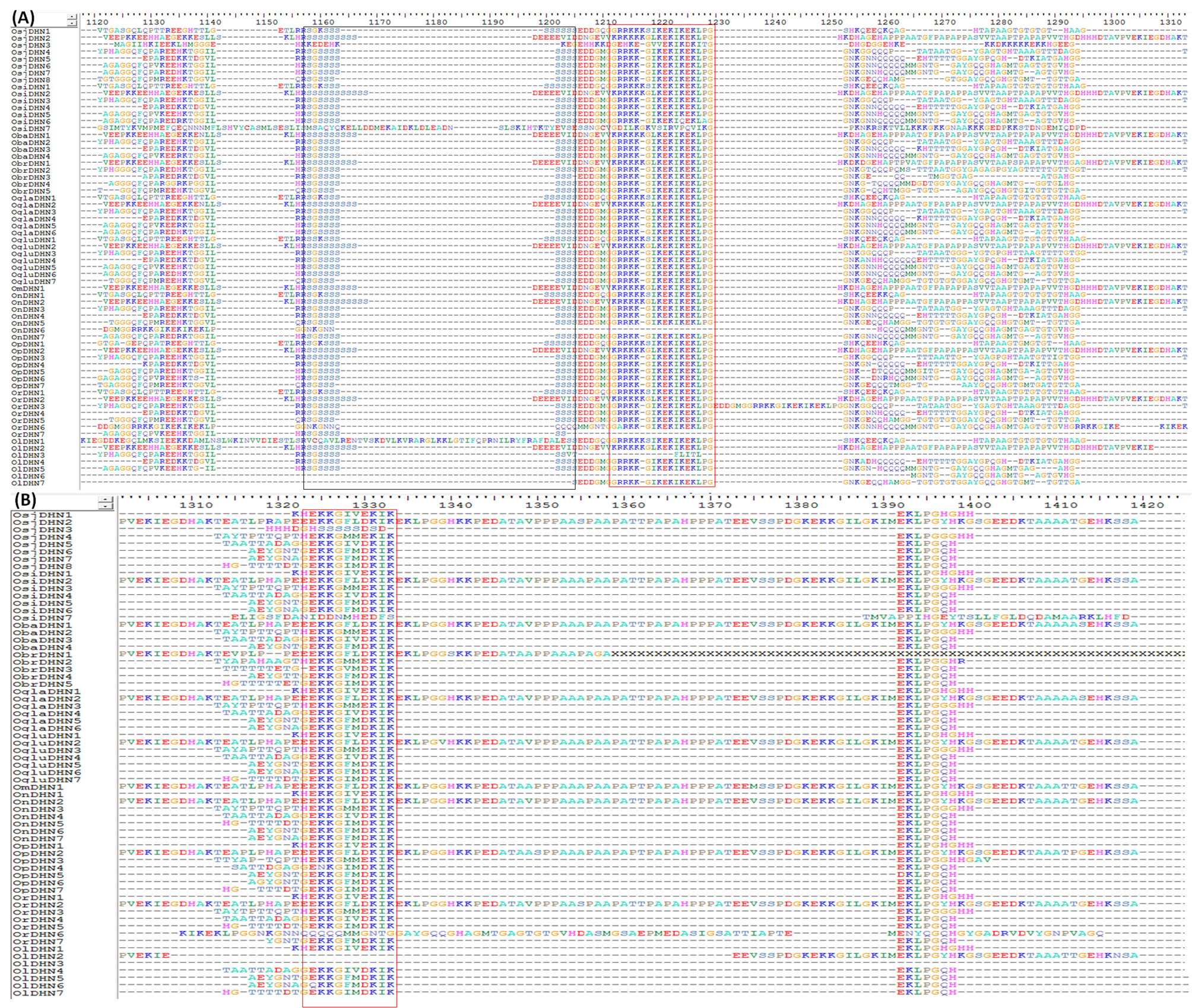

Supplement: S2 Fig — The alignment was performed using ClustalX. The conserved K Y and S segments are highlighted. (TIF) [file pone.0176399.s002.tif]

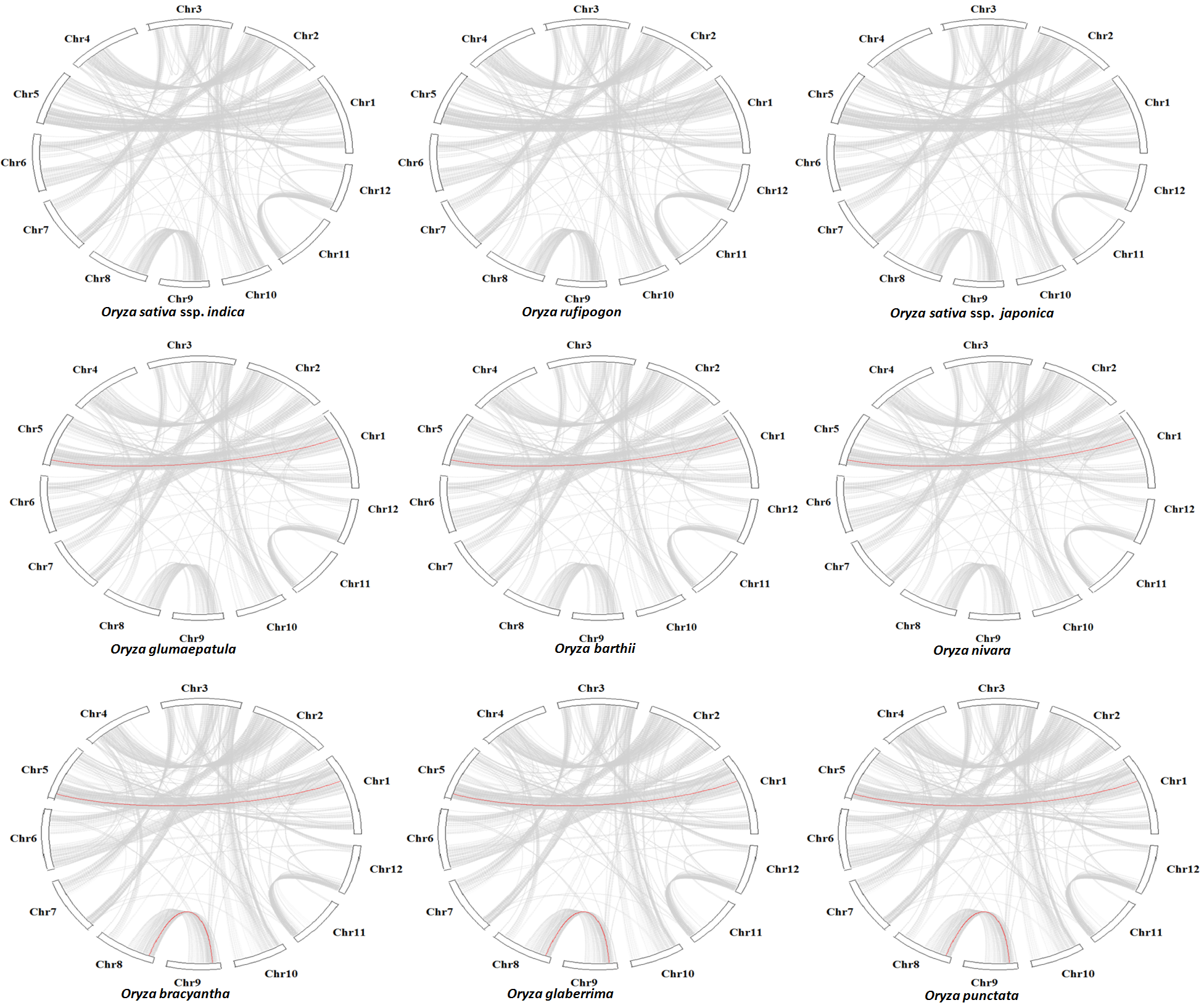

Supplement: S3 Fig — (TIF) [file pone.0176399.s003.tif]

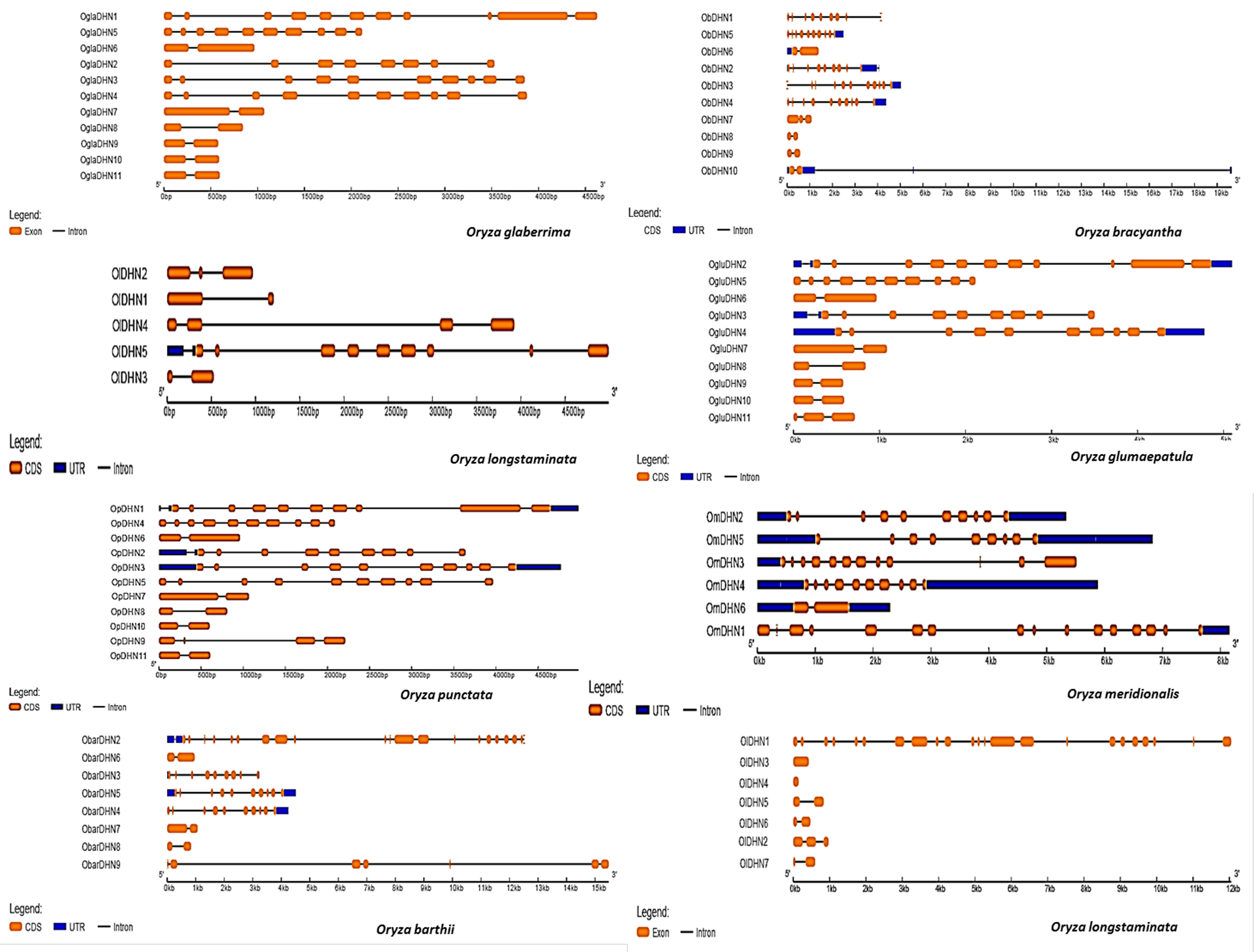

Supplement: S5 Fig — (TIF) [file pone.0176399.s005.tif]
