# Supplementary material for: Genome-wide analysis of rice dehydrin gene family: Its evolutionary conservedness and expression pattern in response to PEG induced dehydration stress
Source: PLoS One. 2017 May 1;12(5):e0176399. doi: 10.1371/journal.pone.0176399 (PMC5411031; doi:10.1371/journal.pone.0176399)
Supplement: S1 Table — (DOC) [file pone.0176399.s006.doc]

S1 Table. Forward and reverse primers used for qRT PCR analysis.

| **Primer Name** | **Forward sequence** | **Reverse sequence** |
| --- | --- | --- |
| OsjDHN1 | CAGATACCGCCTGTCGCCTCCTG | GTCGTCGAGTTGCTTCCTCCTCAG |
| OsjDHN2 | GAGGTGATCGATGACAACGGCGAG | GAGCTGGCGTGGGCGCGGCCGTCAC |
| OsjDHN3 | GAAGGAAGACGAGCACAAGAAGGAG | CTTCTCCTTGTGCTCGCCGCCGTC |
| OsjDHN4 | CAGCACGGAGCTGGTGGACTAG | CATGGTGTCGGGTGTTGTGGTGGC |
| OsjDHN5 | GATGGGAGGAAGGAGGAAGAAG | CGGTGGCGATCTTGGTATCGTGGC |
| OsjDHN6 | CGGTGAAGGAGGAGCACAAGA | GTTGTTGCCCTTGTTGCCGCCG |
| OsjDHN7 | GCGATGGGGATGGGAGGTCATGC | CGTCGTCCTCAGACGACGAGCTG |
| OsjDHN8 | CGATGAGGGAGGAGCACAAGAC | GAGCTTCTCCTTGATCTTCTCCTTG |
