# Supplementary material for: Genome-wide analysis of rice dehydrin gene family: Its evolutionary conservedness and expression pattern in response to PEG induced dehydration stress
Source: PLoS One. 2017 May 1;12(5):e0176399. doi: 10.1371/journal.pone.0176399 (PMC5411031; doi:10.1371/journal.pone.0176399)
Supplement: S2 Table — (DOC) [file pone.0176399.s007.doc]

S2 Table: Synonymous and non-synonymous substitution analysis in rice species

| ***Oryza* species** | ***ds*** | ***dn*** | ***dn*/*ds* ratio** |
| --- | --- | --- | --- |
| *Oryza nivara* | 1.81 | 1.53 | 0.8453 |
| *Oryza glaberrima* | 1.74 | 1.39 | 0.79885 |
| *Oryza sativa* ssp*. indica* | 1.84 | 1.42 | 0.77174 |
| *Oryza rufipogon* | 1.81 | 1.5 | 0.82873 |
| *Oryza sativa* ssp*. japonica* | 1.82 | 1.7 | 0.93407 |
| *Oryza longistaminata* | 0.66 | 0.48 | 0.72727 |
| *Oryza barthii* | 1.79 | 1.16 | 0.64805 |
| *Oryza glumaepatula* | 1.16 | 1.42 | 1.22414 |
| *Oryza brachyantha* | 1.56 | 1.51 | 0.96795 |
| *Oryza punctata* | 1.63 | 1.51 | 0.92638 |
