# Supplementary material for: Genome-wide analysis of rice dehydrin gene family: Its evolutionary conservedness and expression pattern in response to PEG induced dehydration stress
Source: PLoS One. 2017 May 1;12(5):e0176399. doi: 10.1371/journal.pone.0176399 (PMC5411031; doi:10.1371/journal.pone.0176399)
Supplement: S3 Table — (DOC) [file pone.0176399.s008.doc]

***S3 Table: Comparison of regulatory elements in eight DHNs of Oryza sativa ssp japonica.***

| ***cis*-element** | ***OsjDHN1*** | ***OsjDHN2*** | ***OsjDHN3*** | ***OsjDHN4*** | ***OsjDHN5*** | ***OsjDHN6*** | ***OsjDHN7*** | ***OsjDHN8*** | **Function** |
| --- | --- | --- | --- | --- | --- | --- | --- | --- | --- |
| **ABRE** | 0 | 4 | 4 | 1 | 7 | 8 | 8 | 1 | Abscisic acid responsiveness |
| **DRE** | 0 | 2 | 2 | 0 | 0 | 1 | 1 | 2 | Drought and cold responsiveness |
| **LTR** | 0 | 0 | 0 | 0 | 0 | 0 | 1 | 1 | Low0temperature responsiveness |
| **TCA element** | 1 | 1 | 1 | 0 | 0 | 0 | 3 | 0 | Salicylic acid responsiveness |
| **MBS** | 2 | 0 | 1 | 0 | 0 | 0 | 2 | 1 | MYB-binding sites |
| **CGTCA** | 1 | 2 | 1 | 1 | 0 | 2 | 2 | 1 | Me-JA responsive |
| **TGACG** | 1 | 2 | 1 | 1 | 0 | 2 | 2 | 1 | Me-JA responsive |
| **GARE** | 1 | 0 | 0 | 0 | 0 | 0 | 2 | 0 | Gibberellin-responsive |
| **CE03** | 0 | 1 | 0 | 0 | 0 | 1 | 0 | 0 | ABA & VP1 responsive |
| **EIRE** | 0 | 0 | 0 | 0 | 0 | 0 | 0 | 0 | Elicitor responsive |
| **P0Box** | 0 | 2 | 0 | 0 | 0 | 1 | 0 | 0 | Gibberellin responsive |
| **5UTR Py0rich** | 2 | 1 | 1 | 1 | 0 | 0 | 0 | 0 | Biotic stress responsive |
| **GC motif** | 0 | 1 | 3 | 2 | 0 | 1 | 0 | 1 | Enhancer like, anoxic specific inducibility |
| **ARE** | 1 | 1 | 0 | 1 | 0 | 0 | 2 | 0 | Essential for anaerobic induction |
| **MYCR** | 0 | 0 | 1 | 1 | 0 | 0 | 0 | 1 | Water deficit, ABA responsive |
